# Effects of fertilizer application on the bacterial community and weathering characteristics of typical purple parent rocks

**DOI:** 10.3389/fmicb.2024.1514646

**Published:** 2025-01-07

**Authors:** Xuan Wang, Jixia Zhao, Chunpei Li, Limei Deng, Rongyang Cui, Tao Zhou, Zakir Hussain, Gangcai Liu

**Affiliations:** ^1^Key Laboratory of Mountain Surface Processes & Ecological Regulation, Institute of Mountain Hazards and Environment, Chinese Academy of Sciences, Chengdu, China; ^2^University of Chinese Academy of Sciences, Beijing, China; ^3^College of Resources and Environment, Yunnan Agricultural University, Kunming, China

**Keywords:** bacterial community composition, bioweathering, fertilization, rock weathering, weathered products

## Abstract

**Introduction:**

Rock weathering is a fundamental process that shapes Earth’s topography, soil formation, and other surface processes. However, the mechanisms underlying the influence of fertilizer application on weathering remain poorly understood, especially with respect to bacterial intervention.

**Methods:**

In this study, purple parent rocks from Shaximiao Group (J_2_s) and Penglaizhen Group (J_3_p) were selected to investigate the effects of fertilizer application on the bacterial community and weathering characteristics of these rock by leaching experiment.

**Results:**

The results revealed that: fertilizer application, especially when at high levels, greatly altered the abundance, diversity and composition of the bacterial community in weathered products. Through redundancy analysis, a decrease in pH and increases in available nutrients (AN and AP) resulting from fertilizer application were identified as the key factors driving changes of bacterial community composition in weathered products. Moreover, fertilizer application promotes the physical and chemical weathering of the parent rocks to some extent. This is especially true for the chemical weathering of J_2_s. Structural equation model indicated that fertilizer application affects weathering through multiple pathways by affecting the chemical properties (pH, C:N and AP), specific bacterial genera (IMCC26256, *Ramlibacter*, and *Nitrosospira*), and bacterial community composition of weathered products.

**Discussion:**

Our study links weathering characteristics with chemical properties and bacterial community changes of weathered products after fertilizer application, which plays a key role in controlling and predicting dynamic changes of rock weathering in space and time. It is helpful to further understand the law of human activities affecting the surface processes.

## 1 Introduction

Weathering is a fundamental process of the land surface, which directly affects the topography, soil formation, material circulation and other surface processes (Balland et al., 2010; Eppes et al., 2002; Xi et al., 2018). Moreover, the consumption of atmospheric CO_2_ over geological timescales plays a crucial role in global climate change (Goldsmith et al., 2010; Them et al., 2017). In this process, substances (rocks, minerals, etc.) disintegrate (physical weathering), decompose (chemical weathering) and form new substances (Fang et al., 2023), which are influenced by various natural and anthropogenic factors (Jin and Gan, 2013; Pawlik, 2013). However, since the middle of the 20th century, anthropogenic activities have caused unprecedented and rapid changes (Lewis and Maslin, 2015) in land use (Song et al., 2018), biodiversity (Isbell et al., 2017), environmental conditions (Marvel et al., 2019), and material cycling (Austin et al., 2013). Notably, recent studies have estimated that anthropogenic activities contribute between 16 and 40% of weathering processes on the basis of mass balance calculations (Huang et al., 2016; Yu et al., 2016).

Fertilizer application is an important activity in agricultural production, and with the continuous increase in the global population, agricultural activities have intensified, which has led to increased fertilizer usage. According to the official website of the Food and Agriculture Organization of the United Nations, the global consumption of N, P_2_O_5_, and K_2_O fertilizers reached a staggering 201 million tons in 2020. This figure reflects a remarkable 49% increase compared with 2000. Long-term and excessive fertilizer application affects the soil nutrient content, pH, cation exchange capacity and other properties. Changes in soil chemical properties lead to changes in the process and rate of weathering. For example, the introduction of organic matter through livestock organic fertilizers has been shown to inhibit the weathering of carbonate rocks by increasing CO_2_ concentrations and increasing the pH (Song et al., 2017b). Conversely, the soil acidification caused by the application of chemical fertilizers, especially ammonium nitrogen fertilizer (Guo et al., 2010; Bibi et al., 2014; Raza et al., 2020), leads to the leaching of salt group ions and the activation of aluminum (Bibi et al., 2014; Zhao et al., 2022), while causing a decrease in rock strength and disruption of the rock structure. This process contributes to the acceleration of rock physicochemical weathering. Biological weathering occurs when physicochemical weathering is assisted by biological processes. Bacterial weathering, as a significant pathway in bioweathering, plays a vital role in rock weathering (Sel and Binal, 2021). Bacteria can accelerate mineral weathering reactions through various mechanisms, including the production of organic acids and metal–ligand carriers, alteration of redox conditions, and the formation of biofilms (Wu, 2007; Mao et al., 2017; Chen et al., 2022). Increasing evidence supports the notion that bacteria profoundly mediate mineral weathering processes. Fertilizer application is known to cause the changes of bacterial abundance, diversity and community composition in soil (Yuan et al., 2013; Leff et al., 2015; Zeng et al., 2016; Ling et al., 2017; Yu et al., 2019). This change is closely related to the soil chemical properties affected by fertilizer application. Consequently, studying bacteria under different fertilization conditions is pivotal for understanding the complexities of weathering processes.

Weathering is a slow process that is difficult to observe. But owing to their easy weathering characteristics, purple parent rocks are prone to disintegration under the influence of environmental factors and anthropogenic activities. Therefore, they represent an ideal material for studying weathering processes. Through field observations and simulation experiments, scholars have reported that the weathering disintegration thickness of purple parent rocks can reach 11.2 ~ 19.6 mm.yr.^−1^ on average (Zhu et al., 2008). In recent years, we have determined the rates of physical weathering of a variety of purple parent rocks under different influencing factors through a series of indoor and outdoor experiments (Zhang et al., 2016; Zhao et al., 2018). These studies mainly addressed natural factors such as water, heat and acid deposition. There are few studies concerned with anthropogenic activity influence-fertilizer application. Therefore, the objective of this study was to comprehensively examine the degree of weathering and the mechanisms underlying the effects of fertilization on typical purple parent rocks under various fertilization conditions. The elucidation of these relationships will contribute to the development of sustainable strategies for purple soil regions, ensuring their long-term productivity and environmental integrity.

## 2 Materials and methods

### 2.1 Field sites

The experimental site is located at the Yanting Purple Soil Agroecology Experimental Station of the Chinese Academy of Sciences (N 31°16′52″ E105°27′20″), at an altitude of 420 m. The average annual temperature in the experimental area is 17.3°C. The average annual rainfall is 880.7 mm. The predominant cropping system involves winter wheat and summer corn cultivation.

### 2.2 Rock sample preparation and experiment description

Two representative purple parent rocks, namely, Shaximiao Group (J_2_s) (N 29°46′04″ E 104°48′42″) and Penglaizhen Group (J_3_p) (N 31°16′52″ E 105°27′20″), which account for the largest exposed area in the Sichuan Basin, were selected as the experimental materials. To ensure the reliability and comparability of the experimental data, parent rocks of the same lithological type were sourced from identical sources and exhibited consistent coloration and degrees of weathering. The parent rock samples were subsequently naturally dried, followed by crushing and sieving to obtain rock fragments with five distinct particle size fractions: > 60 mm, 40–60 mm, 20–40 mm, 10–20 mm, and 5–10 mm. The mineral composition (Supplementary Table S1), major elemental content (Supplementary Table S2) and chemical properties (Supplementary Table S3) of the original parent rocks were determined.

Leaching columns with a diameter of 160 mm and a height of 400 mm were prepared for the experiment. The columns were filled with 600 g of rock fragments of each particle size fraction, totaling 3,000 g. Larger rock fragments were positioned at the bottom of the column, whereas smaller fragments were placed at the top. The experiment was set up with two variables: the application of fertilizer type and level. Nitrogen fertilizer (NH_4_HCO_3_) applied alone and combined application of nitrogen, phosphorus and potassium fertilizers (NH_4_HCO_3_, NH_4_H_2_PO_4_ and KCl) were selected as the two types of fertilizer. The amount of fertilizer was determined according to the conventional fertilization amount (for the wheat planting period, N-P_2_O_5_-K_2_O = 130-90-36 kg · ha^−1^ · a^−1^; for the corn planting period, N-P_2_O_5_-K_2_O = 150-90-36 kg · ha^−1^ · a^−1^). The conventional fertilization amount was considered the 100% fertilizer level, and the experiment included four levels: 0, 100, 200 and 300%. Therefore, the experiment consisted of seven treatments: (i) control group without fertilizer (CK); (ii) 100% N fertilizer treatment (N1); (iii) 200% N fertilizer treatment; (iv) 300% N fertilizer treatment (N1); (v) 100% NPK fertilizer treatment (NPK1); (vi) 200% NPK fertilizer treatment; and (vii) 300% NPK fertilizer treatment. The fertilizers were dissolved in 50 mL of deionized water and uniformly applied to the surface layer of the leaching columns. The leaching columns were installed in a field in June 2021 for the natural rainfall leaching experiments. The timing of fertilization was based on the local fertilization schedule in the months of June and October each year. After each rainfall event, the leached solutions were collected from the leaching columns. The weathered products of the parent rock were collected in September 2023. The samples were subjected to bacterial community analysis at −4°C, and physical and chemical properties were assessed after the samples were subjected to room temperature drying.

### 2.3 Chemical properties of weathered products

The weight of the <2 mm particles in the weathered products was measured via dry sieving, and the chemical characteristics of these particles were determined after drying at 65°C. The pH was determined via a potentiometric method at a water-to-soil ratio of 2.5:1. The cation exchange capacity (CEC) was determined via cobalt hexachloride leaching spectrophotometry (Renault et al., 2009). The total nitrogen (TN) and soil organic carbon (SOC) contents were determined via a combustion elemental analyzer. The contents of total phosphorus (TP), total potassium (TK), and other conserved elements were determined via X-ray fluorescence spectroscopy (XRF). Available nitrogen (AN) was determined via the alkaline hydrolysis diffusion method. Available phosphorus (AP) was determined via the sodium bicarbonate extraction molybdenum antimony colorimetric method. Available potassium (AK) was determined via ammonium acetate extraction flame photometry. The sample mineral composition was determined via X-ray diffraction (XRD) analysis (Sanchez and Gunter, 2006).

### 2.4 DNA extraction, PCR amplification, and high-throughput sequencing

The total genomic DNA of weathered product samples was extracted via an OMEGA Soil DNA Kit (OmegaBio-Tek, Norcross, GA, United States). Bacterial 16S rRNAV3-V4 region-specific primers were selected for PCR amplification, and according to the selected 16S V4 region, PCR amplification was carried out via the primers 338F (5′-barcode+ACTCCTACGGGGAGGCAGCA-3′) and 806R (5′-GGACTACHVGGGTWTCTAAT-3′). The amplification products were subjected to 2% agarose gel electrophoresis, and the target fragments were excised and then recovered via an Axygen Gel Recovery Kit. The PCR products were quantified on a microplate reader (BioTek, FLx800) via the Quant-iT PicoGreen dsDNA Assay Kit. After the quantification step, the samples were mixed according to the amount of data required for each sample. Sequencing was performed via the NovaSeq-PE250 pattern of the Illumina MiSeq platform at Shanghai Personal Biotechnology Co., Ltd. (Shanghai, China).

### 2.5 Evaluation indices of weathering

The degree of chemical weathering is often determined through the analysis of geochemical parameters. The chemical index of alteration (*CIA*), which effectively captures the extent of weathering by considering various elements involved in the weathering process and exhibiting a monotonic response to silicate weathering (Nesbitt et al., 1996), has been widely used to assess the degree of rock weathering. The *CIA* is calculated as follows:


(1) $$CIA=\frac{{Al}_{2}O_{3}}{{Al}_{2}O_{3}+{Na}_{2}O+K_{2}O+{CaO}^{*}}\times 100$$

where oxides are presented in molar units. CaO^*^ in Equation 1 is the CaO content in silicate; if n (Na_2_O) > n (CaO), then n (CaO) = n (CaO*), if n (Na_2_O) < n (CaO), then n (Na_2_O) = n (CaO*).

A particle size of <2 mm is considered as the soil-forming standard for purple parent rock. The mass fraction of weathered products of <2 mm particle size was used as the physical weathering index to determine the soil formation rate (*SFR*) (Soil Research Office of Chinese Academy of Sciences Chengdu Branch, 1991).

The calculation of the *SFR* is performed as follows:


(2) $$SFR=\frac{m-m_{1}}{m}\times 100\%$$

where: *m* in Equation 2 is the total mass of the parent rock in the lysimetric column (3,000 g), and where m_1_ is the mass of weathered products with >2 mm grain size at the end of the experiment.

### 2.6 Statistical analysis

Analysis of variance (ANOVA) and Pearson’s correlation analysis were performed in SPSS 26 to statistically analyze the experimental results. Microbiome bioinformatics was performed with QIIME 2019.4 according to official tutorials. Permutational multivariate analysis of variance (PERMANOVA) was performed with R 4.3.2 to evaluate the differences in bacterial community composition. Redundancy analysis (RDA) was performed by CANOCO 5 to evaluate the effects of the chemical properties of the weathered products on the total bacterial community composition. The above data were visualized in Origin 2021 and R 4.3.2. Structural equation models (SEMs) of the relationships between fertilization conditions, chemical properties, bacteria and degree of weathering were constructed via SMARTPLS 3 (Armonk, United States).

## 3 Results

### 3.1 Chemical properties of weathered products

As shown in Table 1, the chemical properties of the weathered products changed to different degrees under the different fertilizer treatments. For J_2_s, the pH, CEC and SOC content of all of the fertilizer treatments were significantly lower than those of the CK treatment (except for the SOC content under the NPK1 fertilizer treatment) (*p <* 0.05). The pH decreased with increasing fertilizer level under the same fertilizer type. The order of magnitude of pH was NPK < N < CK. Conversely, the AN and AK contents in all of the fertilizer treatments were greater than those in the CK treatment, with corresponding increases as the fertilizer level increased. The order of magnitude of the AN and AK contents was CK < N < NPK. The AN content under all the fertilizer treatments and the AK content under the NPK fertilizer treatments were significantly greater than those under the CK treatment (*p <* 0.05). The contents of TP and AP in the NPK fertilizer treatments were significantly greater than those in the CK treatment (*p <* 0.05), with corresponding increases as the fertilizer level increased. The content of TP in the N fertilizer treatment was not significantly different from that in the CK treatment (*p >* 0.05), but the AP content was significantly lower than that of the CK treatment (*p <* 0.05). The contents of TN and TK did not significantly differ among the treatments (*p >* 0.05). The chemical properties of J_3_p were less sensitive to fertilizer treatments than those of J_2_s were. There were no significant changes in the chemical properties (*p >* 0.05), except for the contents of TP and available nutrients, which were significantly greater under the NPK fertilizer treatments than under the CK treatment (*p <* 0.05).

**Table 1:** Weathered product chemical properties under different treatments.

Parent rock	Treatment	pH	CEC	TN	SOC	TP	TK	AN	AP	AK
J_2_s	CK	9.79 ± 0.03a	20.08 ± 0.18a	0.03 ± 0.00a	0.22 ± 0.09a	0.09 ± 0.01c	3.20 ± 0.01ab	15.53 ± 3.39d	16.19 ± 8.00d	99.92 ± 0.42d
	N1	9.33 ± 0.06b	19.90 ± 0.30b	0.03 ± 0.00a	0.15 ± 0.03b	0.09 ± 0.01c	3.21 ± 0.02ab	25.26 ± 0.33c	11.06 ± 2.68d	102.58 ± 7.84d
	N2	9.27 ± 0.05bc	19.94 ± 0.23ab	0.03 ± 0.00a	0.14 ± 0.02b	0.08 ± 0.00c	3.19 ± 0.02b	27.38 ± 3.34c	9.28 ± 3.84d	103.60 ± 0.73d
	N3	9.11 ± 0.05c	19.20 ± 0.20b	0.03 ± 0.00a	0.15 ± 0.04b	0.10 ± 0.02c	3.25 ± 0.03a	60.61 ± 7.68b	14.03 ± 8.49d	111.88 ± 21.99d
	NPK1	8.79 ± 0.09d	18.88 ± 0.51b	0.04 ± 0.00a	0.23 ± 0.01a	0.19 ± 0.02b	3.17 ± 0.02b	58.70 ± 6.41b	38.70 ± 2.01c	174.47 ± 7.07c
	NPK2	8.55 ± 0.16e	19.11 ± 0.51b	0.03 ± 0.00a	0.14 ± 0.01b	0.24 ± 0.02a	3.24 ± 0.05ab	68.21 ± 7.60b	55.17 ± 3.57b	245.00 ± 16.08b
	NPK3	8.52 ± 0.15e	19.35 ± 0.22b	0.03 ± 0.00a	0.14 ± 0.01b	0.24 ± 0.01a	3.24 ± 0.02ab	96.26 ± 5.03a	67.60 ± 4.66a	309.57 ± 18.44a
J_3_p	CK	9.05 ± 0.01ab	19.21 ± 0.48a	0.04 ± 0.00a	0.20 ± 0.06a	0.08 ± 0.00d	2.89 ± 0.03a	19.59 ± 1.07d	7.58 ± 1.27d	115.40 ± 3.00d
	N1	9.04 ± 0.03ab	18.57 ± 0.24a	0.03 ± 0.00a	0.12 ± 0.01a	0.08 ± 0.00d	2.95 ± 0.08a	19.58 ± 5.34d	6.31 ± 1.13d	110.00 ± 6.10d
	N2	9.06 ± 0.02ab	18.82 ± 0.80a	0.04 ± 0.01a	0.15 ± 0.03a	0.07 ± 0.00d	2.91 ± 0.07a	21.39 ± 3.57 cd	5.40 ± 0.85d	106.75 ± 5.75d
	N3	9.02 ± 0.01ab	17.71 ± 0.30a	0.03 ± 0.00a	0.14 ± 0.03a	0.08 ± 0.00d	2.83 ± 0.04a	24.88 ± 3.53 cd	7.72 ± 2.33d	98.25 ± 1.52d
	NPK1	9.08 ± 0.01a	17.59 ± 0.61a	0.03 ± 0.00a	0.17 ± 0.03a	0.15 ± 0.00c	2.85 ± 0.05a	30.06 ± 3.94c	35.15 ± 1.33c	177.07 ± 7.97c
	NPK2	9.06 ± 0.05ab	17.32 ± 1.13a	0.04 ± 0.00a	0.17 ± 0.02a	0.25 ± 0.01b	2.86 ± 0.09a	41.04 ± 3.90b	60.98 ± 6.01b	275.4 ± 13.31b
	NPK3	8.99 ± 0.03b	17.56 ± 0.85a	0.04 ± 0.00a	0.15 ± 0.02a	0.40 ± 0.02a	2.99 ± 0.04a	52.12 ± 4.40a	96.78 ± 12.52a	350.7 ± 10.54a

CK=No fertilizer; N1 = 280 kg · ha^−1^ · a^−1^ N; N2 = 560 kg · ha^−1^ · a^−1^ N; N3 = 840 kg · ha^−1^ · a^−1^ N; NPK1 = N-P_2_O_5_-K_2_O: 280-180-72 kg · ha^−1^ · a^−1^; NPK2 = N-P_2_O_5_-K_2_O: 560-360-144 kg · ha^−1^ · a^−1^; NPK3 = N-P_2_O_5_-K_2_O:840-540-216 kg · ha^−1^ · a^−1^; CEC(cmol.kg^−1^)—Cation exchange capacity; TN(%)—Total nitrogen; SOC(%)—Soil organic carbon; TP(%)—Total phosphorus; TK(%)—Total potassium; AN(%)—Available nitrogen; AP(%)—Available phosphorus; AK(%)—Available potassium. Numbers depict means ± standard deviations (*n* = 3), and different lowercase letters indicate that there were significant differences among all treatments (*p* < 0.05).

### 3.2 Bacterial abundance, diversity and community composition of weathered products

**Figure 1:**
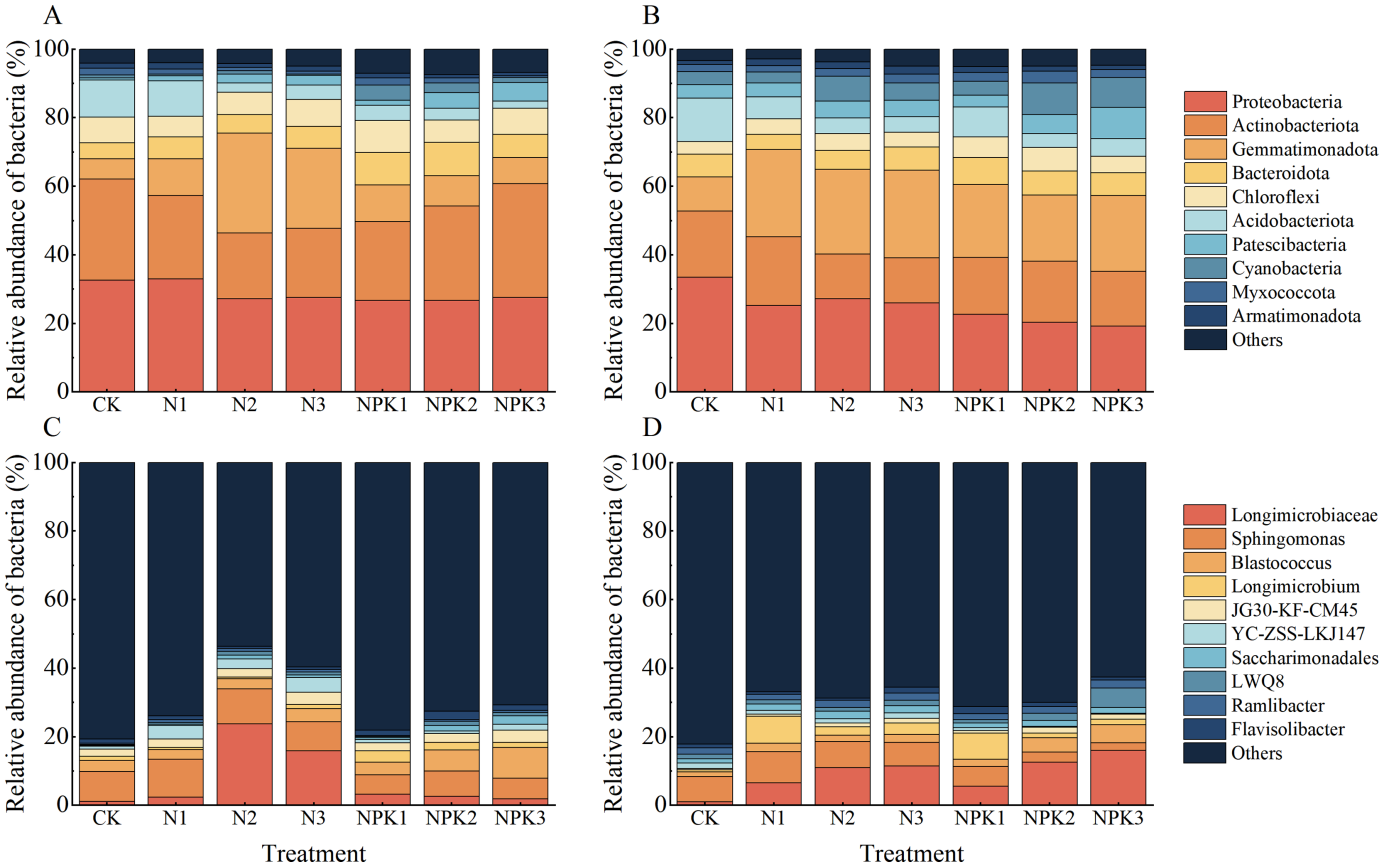
Distribution of bacteria in weathered products under different treatments. **(A)** The dominant phyla of the purple parent rock of J_2_s. **(B)** The dominant phyla of the J_3_p purple parent rock. **(C)** The dominant genus of the purple parent rock of J_2_s. **(D)** The dominant genus of the J_3_p purple parent rock.

A total of 3,923,050 sequences were identified from all samples via Illumina® MiSeq sequencing, and 3,293,394 valid sequences were retained after quality filtering. The sequences were analyzed and identified as belonging to 39 phyla, 129 classes, 337 orders, 559 families, 1,164 genera and 2,780 species. Among them, the dominant phyla were Proteobacteria, Actinobacteriota, Gemmatimonadota, Bacteroidota, Chloroflexi and Acidobacteriots, which collectively accounted for 73.90 to 90.95% of the entire bacterial community. Sixteen genera were identified as abundant (with relative abundances greater than 0.05% in all of the treatments and mean values exceeding 1%). The most abundant genera were *Longimicrobiaceae, Sphingomonas, Blastococcus, Longimicrobium* and JG30-KF-CM45, and the number of sequences of these genera accounted for 23.93% ~ 54.81% of the total number of sequenced genera (Figure 1).

**Figure 2:**
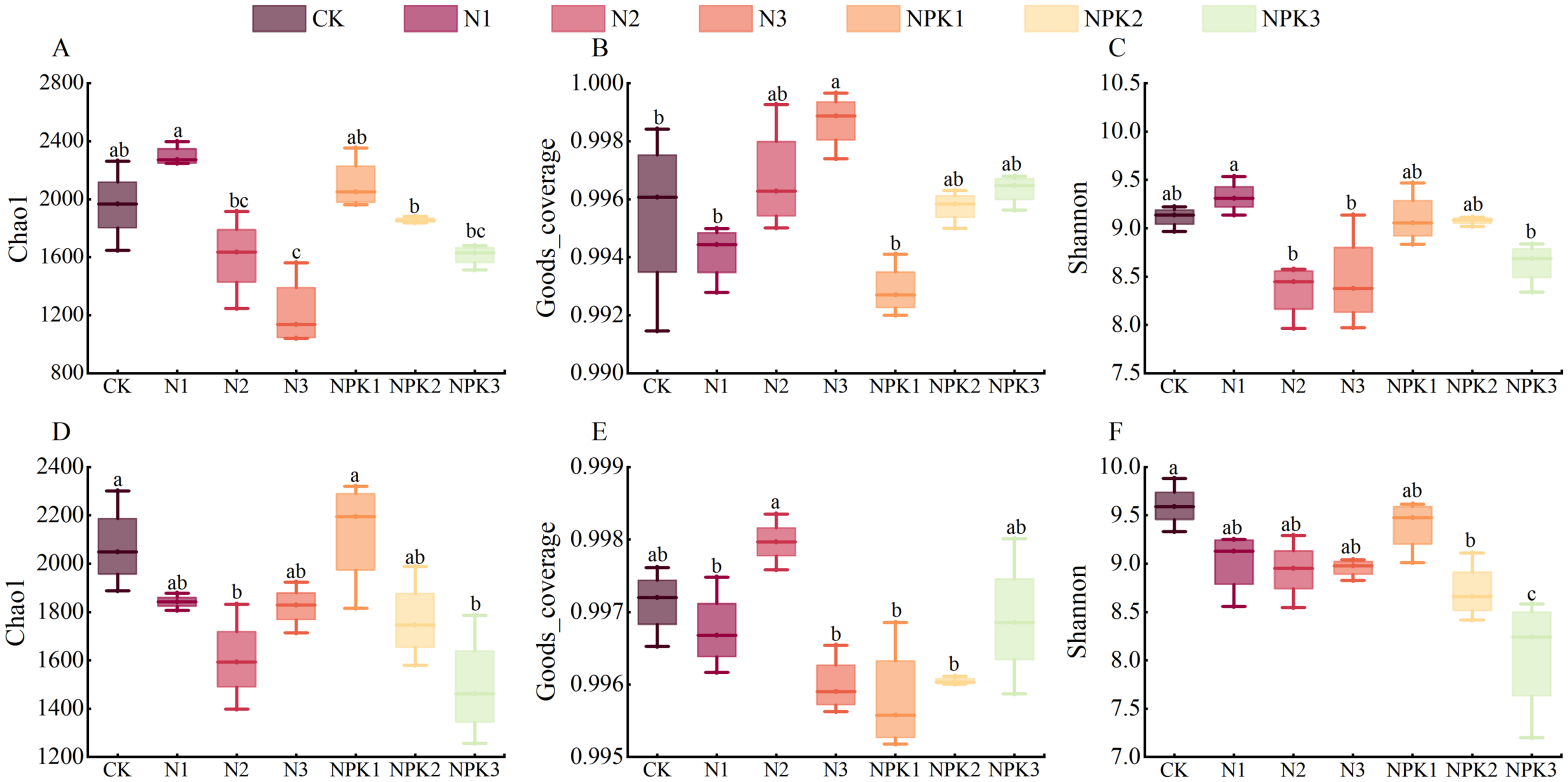
The *α* diversity index of the bacteria in the weathered products under different fertilization treatments. **(A)** Chao1 of the J_2_s purple parent rock. **(B)** Goods_coverage index of the J_2_s purple parent rock. **(C)** Shannon of J_2_s the purple parent rock. **(D)** Chao1 of the J_3_p purple parent rock. **(E)** Goods_coverage index of the J_3_p purple parent rock. **(F)** Shannon index of the J_3_p purple parent rock. Different lowercase letters indicate that there were significant differences among the treatments (*p* < 0.05).

For subsequent analysis, rarefaction curves were plotted, which revealed that the sequencing results sufficiently reflected the diversity contained in the sample (Supplementary Figure S1). To assess the alpha diversity of the bacterial community of weathered products, abundance was characterized by the Chao1, coverage was represented by Good’s coverage index, and diversity was determined by the Shannon. The Good’s coverage indices were all greater than 0.99, indicating that the sequencing capability covered most of the characteristics of the bacterial community (Figure 2).

For J_2_s, the Chao1 of the 100% N and NPK fertilizer treatments was greater than that of the CK treatment, although the difference was not statistically significant (*p >* 0.05). The Chao1 of the 200 and 300% N and NPK fertilizer treatments were lower than those of the CK treatment, with a significant decrease observed at the 300% N fertilizer treatment level (*p <* 0.05). Under the same type of fertilizer, the Chao1 tended to decrease with increasing fertilizer level. The pattern of change in the Shannon was the same as that in the Chao1, but there was no significant difference between the fertilizer treatments and the CK treatment. For J_3_p, the Chao1 was consistently lower than that of the CK treatment for the fertilizer treatments (except for the 100% NPK fertilizer treatment), where the Chao1 was significantly lower under the 200% N and 300% NPK fertilizer treatments (*p <* 0.05). Like in J_2_s, the Chao1 decreased with increasing fertilizer level in the NPK fertilizer treatment. The pattern of change in the Shannon was comparable to that in the Chao1.

**Figure 3:**
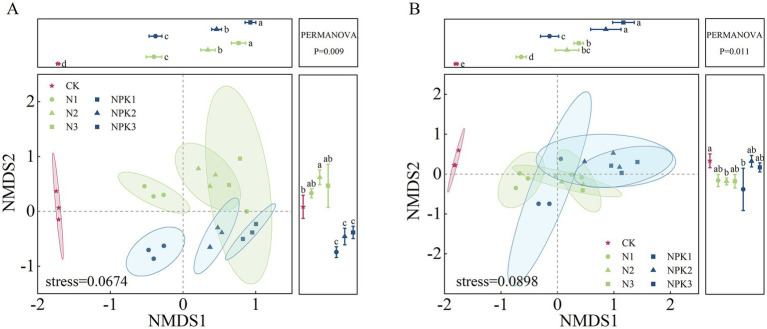
Nonmetric multidimensional scales (NMDS) beta diversity analysis of the bacterial community composition in the weathered products under different treatments. **(A)** J_2_s purple parent rock. **(B)** J_3_p purple parent rock.

Nonmetric multidimensional scaling (NMDS) analysis based on the bray–curtis distance algorithm was conducted to assess the beta diversity of the bacterial community of weathered products. The results revealed distinct differences in the bacterial community among the different fertilizer treatments (Figure 3). The N and NPK fertilizer treatments were distinguished from the CK treatment along NMDS1, whereas the N and NPK treatments were differentiated along NMDS2. Furthermore, clusters representing different fertilizer levels within the same fertilizer types were separated from each other along NMDS1. Overall, the results of PERMANOVA revealed significant differences in community composition among the treatments. Similar patterns were observed for both parent rocks.

**Figure 4:**
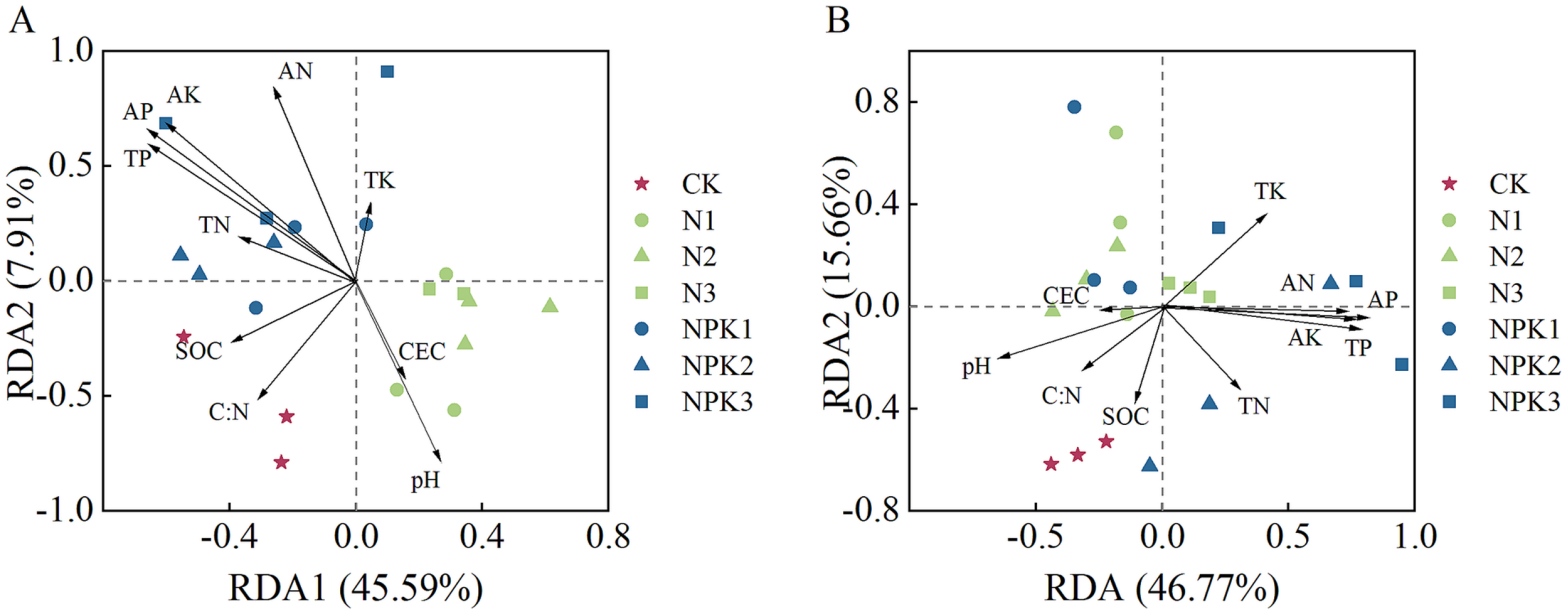
Redundancy analysis (RDA) of the effect of different chemical properties of the bacterial community composition in the weathered products under different treatments. **(A)** J_2_s purple parent rock. **(B)** J_3_p purple parent rock.

Redundancy analysis (RDA) revealed that environmental factors (pH, AP and AN content) played important roles in bacterial community composition of weathered products under the different fertilizer treatments (Figure 4), with AP having the most pronounced effect (Supplementary Table S4).

### 3.3 Effects of fertilizer application on weathering

**Figure 5:**
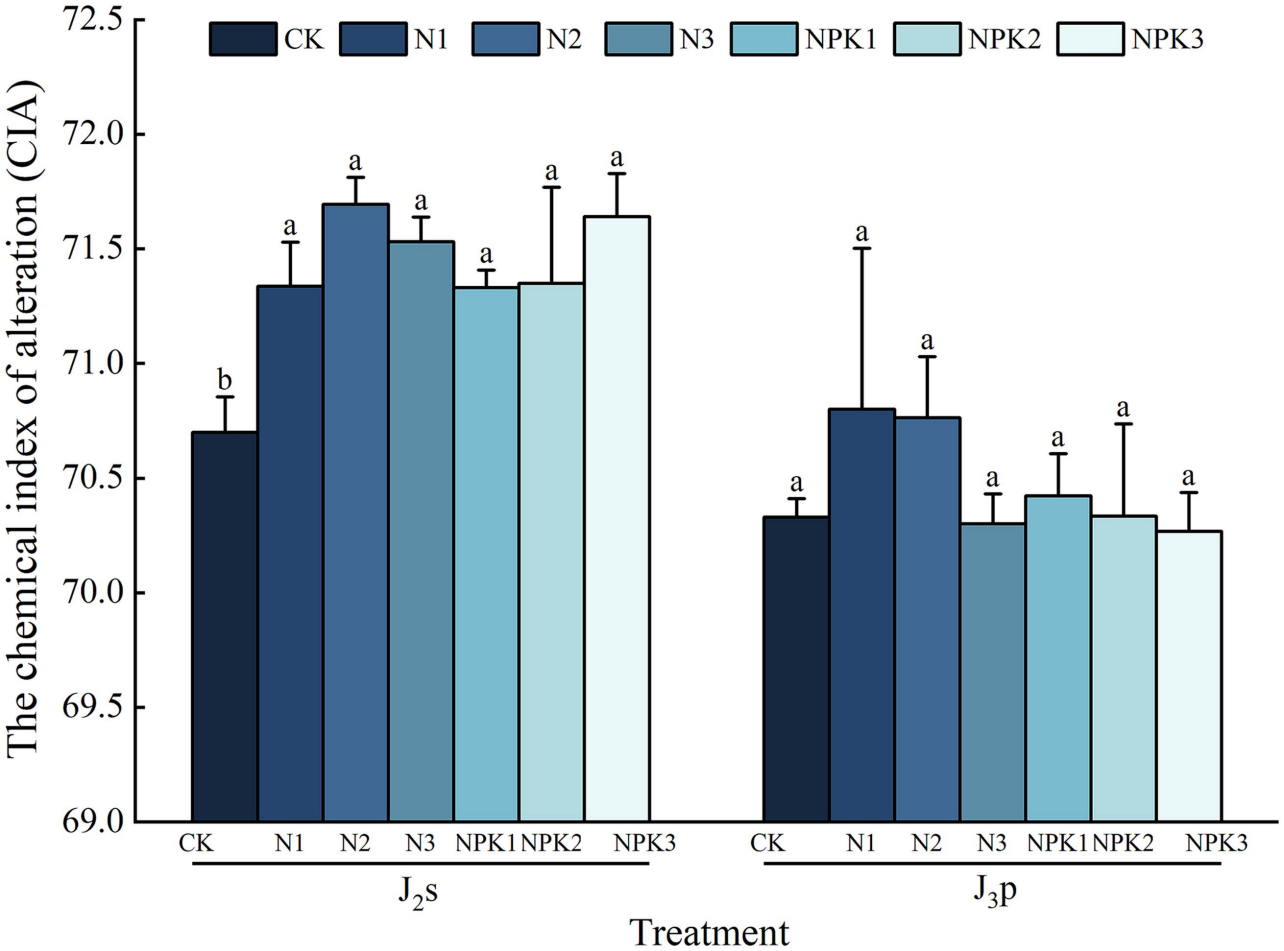
Chemical index of alteration (*CIA*) of the weathered products under different treatments. Values are the means with standard deviations shown by vertical bars (*n =* 3). Different lowercase letters indicate that there were significant differences among all treatments (*p <* 0.05).

The *CIA* revealed that fertilizer application generally promoted chemical weathering of the parent rocks (Figure 5). Specifically, for J_2_s, compared with the CK treatment, all the fertilizer treatments significantly increased the *CIA*, with increases ranging from 0.89 to 1.40% (*p <* 0.05). In contrast, compared with J_2_s, J_3_p presented a lower degree of chemical weathering and a lower sensitivity to fertilizer. Compared with that under the CK treatment, the CIA of the weathered products under the 300% N fertilizer treatment decreased by 0.04%. The *CIA* of the other fertilizer treatments was greater than that of the CK treatment, increasing by 0.13% ~ 0.67%, but there was no significant difference between the treatments (*p >* 0.05). Within the same type of fertilizer, the *CIA* of weathered products tended to decrease with increasing level of fertilizer.

**Figure 6:**
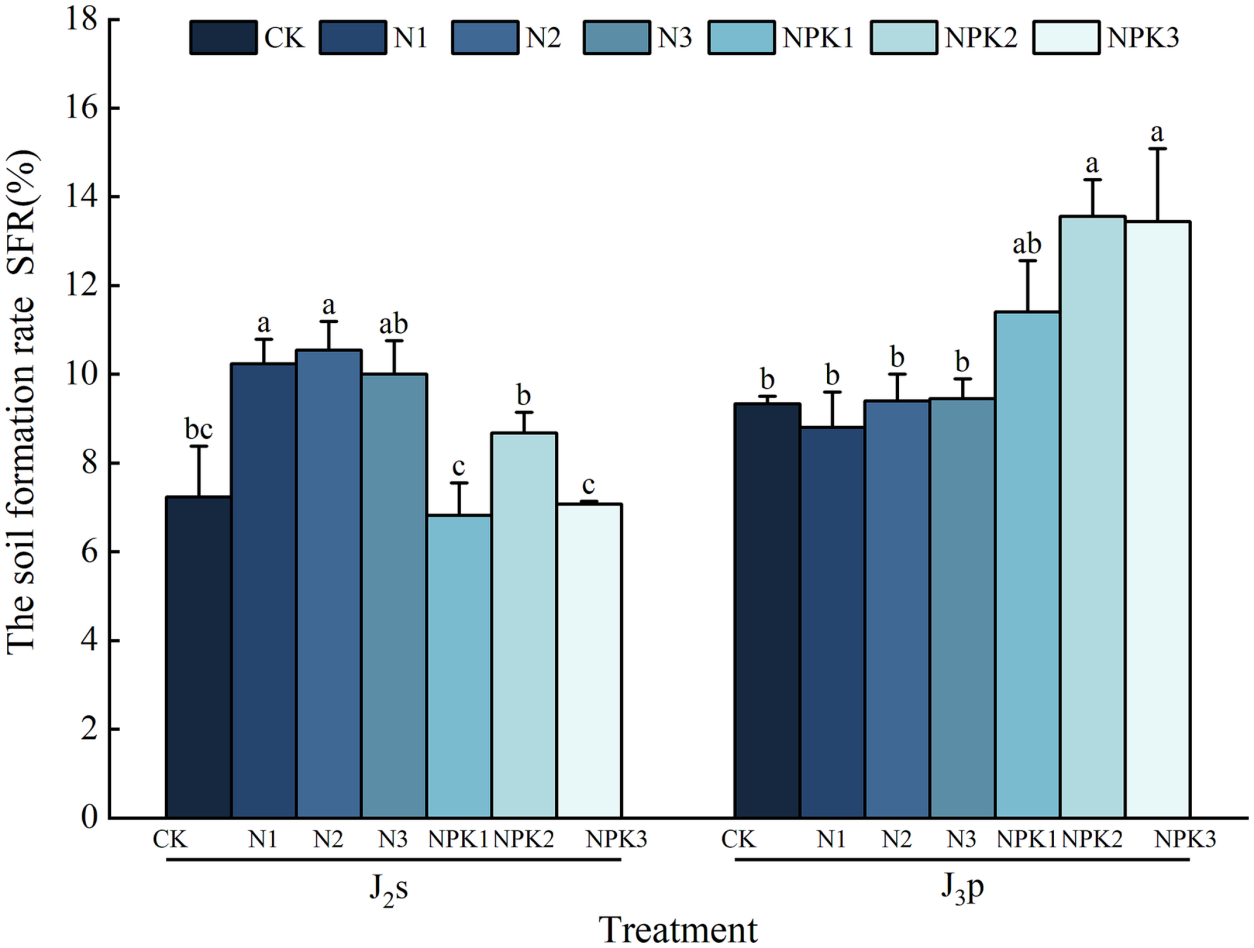
The soil formation rate (*SFR*) of the weathered products under different treatments. Values are means with standard deviations shown by vertical bars (*n* = 3). Different lowercase letters indicate that there were significant differences among all treatments (*p <* 0.05).

The *SFR* was calculated as an index of the degree of physical weathering (Figure 6), and the *SFR* of J_2_s was 6.82% ~ 10.54% under each treatment. Compared with those in the CK treatment, the *SFR* in the N and 200% NPK fertilizer treatments increased by 19.97% ~ 45.78%, with a significant increase under 100 and 200% N fertilizer treatments (*p <* 0.05). However, the *SFR* decreased by 2.15% ~ 5.68% under the 100 and 300% NPK fertilizer treatments, but the differences were not statistically significant (*p >* 0.05).

Compared with the CK treatment, all of the fertilizer treatments (except for the 100% N fertilizer treatment) resulted in an increase in the *SFR*, ranging from 0.71 to 45.24%, in J_3_p. The 200 and 300% NPK fertilizer treatments significantly increased the *SFR* (*p <* 0.05). In contrast, the *SFR* of the 100% N fertilizer treatment decreased by 5.71%, but the difference was not statistically significant (*p >* 0.05). Overall, except for the N fertilizer treatments in J_3_p, the *SFR* of all the treatments for both parent rocks reached their peak values at the 200% fertilizer level.

Multivariate analysis of variance (MANOVA) was conducted to examine the influence of parent rock type and fertilizer conditions on the weathering indices (Table 2). The results revealed a significant effect of parent rock type on the *CIA* (*p <* 0.05). Both parent rock type and fertilizer type had a significant effect on the *SFR* (*p <* 0.05), and their interaction also had a significant effect (*p <* 0.05).

**Table 2:** Multivariate analysis of variance (MANOVA) of rock type, fertilizer type and fertilizer level.

Weathering index	Treatment	DF	MS	F	*p*_value
CIA	Rock type	1	8.980	66.208	0.000
	Fertilizer type	1	0.292	2.150	0.156
	Fertilizer level	2	0.031	0.229	0.797
	Rock × fertilizer type	1	0.090	0.664	0.423
	Rock × fertilizer level	2	0.254	1.871	0.176
	Fertilizer type × fertilizer level	2	0.136	1.001	0.382
	Rock × fertilizer type × fertilizer level	2	0.017	0.124	0.884
SFR	Rock type	1	0.004	33.080	0.000
	Fertilizer type	1	0.000	3.743	0.041
	Fertilizer level	2	0.000	1.335	0.261
	Rock type × fertilizer type	1	0.008	73.714	0.000
	Rock type × fertilizer level	2	0.000	1.214	0.317
	Fertilizer type × fertilizer level	2	0.000	1.502	0.246
	Rock × fertilizer type × fertilizer level	2	0.000	0.166	0.848

### 3.4 Effects of fertilizer-driven biochemical properties on weathering

**Figure 7:**
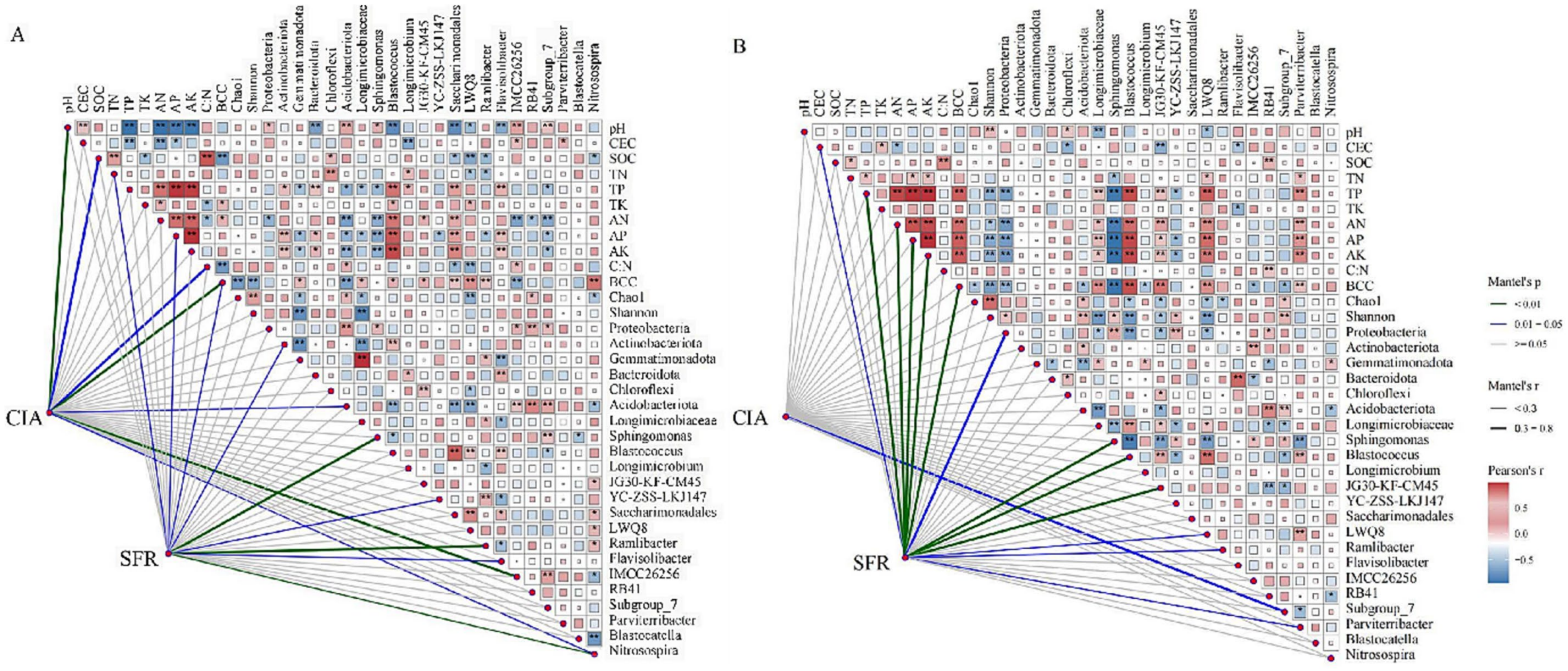
Correlations among weathering evaluation indices (the chemical index of alteration and the soil formation rate), chemical properties, dominant phyla, dominant genera, bacterial abundance, diversity and community composition. **(A)** J_2_s purple parent rock. **(B)** J_3_p purple parent rock. * and ** indicate significances at *p <* 0.05 and *p <* 0.01.

Correlation analyses were conducted to investigate the relationships between chemical properties, bacterial diversity indicators, bacterial community composition (BCC), dominant phyla, dominant genera, and weathering indices. In J_2_s, the *CIA* was significantly negatively correlated with pH, the SOC content, the C:N, and the relative abundance of Acidobacteriota and IMCC26256, and was significantly positively correlated with the relative abundance of *Nitrosospira*. Moreover, a correlation was observed between the bacterial community composition and the *CIA*. The *SFR* was significantly negatively correlated with the contents of TN and AP and the relative abundances of Actinobacteria and *Flavisolibacter* and significantly positively correlated with the relative abundances of *Sphingomonas*, YC-ZSS-LKJ147, *Ramlibacter*, and *Nitrosospira*. The bacterial community composition was also correlated with the *SFR*. Among the factors affecting J_3_p, only the relative abundance of Subgroup_7 was significantly correlated with the *CIA*. The *SFR* was significantly negatively correlated with the CEC and the relative abundances of Proteobacteria and *Sphingomonas* and significantly positively correlated with the contents of TP, AN, AP and AK and the relative abundances of *Blastococcus*, JG30-KF-CM45, LWQ8, *Ramlibacter*, and *Parviterribacter*. Like that in J_2_s, bacterial community composition in J_3_p was also related to the *SFR* (Figure 7). A comparison of the two weathering indices revealed that physical weathering was more sensitive to each factor than chemical weathering.

## 4 Discussion

Fertilizer application profoundly influences the chemical properties of weathered products. The pH decreased after fertilizer application (Table 1). This finding is consistent with the results of previous soil-related studies (Guo et al., 2010; Zhang et al., 2024). Nitrate leaching caused by fertilizer application accounted for 59% ~ 66% of the total acidity in semiarid regions of the U.S.A (Tarkalson et al., 2006). The pH reduction is attributed primarily to the production of protons during the process of ammonium oxidation to nitrite and further to nitrate. The loss of nitrate removes alkaline ions such as calcium and magnesium, consequently causing soil acidification (Raza et al., 2020). Notably, the pH decrease in J_3_p was not significant compared with that in J_2_s because of its higher content of CaCO_3_, which acts as a neutralizing agent (Zhao et al., 2022). Compared with those in the CK treatment, some nutrients significantly increased under the fertilizer treatments, which resulted from the exogenous nutrient inputs from fertilizer application. However, except for TP (under the NPK fertilizer treatments), the contents of the total nutrients did not significantly increase, and even the SOC content was lower than that in the CK treatment. Available nutrients can be adsorbed by clay minerals, such as illite, which adsorbs ammonium nitrogen, and with time, its lattice ion exchange stabilizes available nitrogen into total nitrogen (Heleen et al., 2021). In this study, we used rock as the material, as the specific surface area and clay mineral content are much lower than those of soil, making it difficult to fix the available nutrients, and there were no plant-mediated nutrients, so the total nutrients cannot be increased. In addition, the sole application of chemical fertilizers leads to a decline in the content of SOC, due to its inability to provide organic carbon sources and its ability to alter the composition and activity of the microbial community (Demoling et al., 2007). Specifically, the application of nitrogen fertilizer alone intensifies the activities of ammonia-oxidizing bacteria and nitrifying bacteria (Han et al., 2023), and these microorganisms consume SOC during their metabolic procedures, thus reducing the content of SOC and the C:N (Zhong et al., 2016; Mori et al., 2018).

The characteristics of the bacterial community of weathered products also changed concomitantly with the changes in chemical properties caused by fertilizer application. Consistent with previous research, the bacterial abundance and diversity of weathered products significantly decreased with increasing levels of fertilizer (Yuan et al., 2013; Burns et al., 2015; Zeng et al., 2016; Yu et al., 2019). Correlation analysis revealed that pH, contents of TP and available nutrients play pivotal roles in shaping bacterial diversity (Figure 7), which is consistent with the findings of previous studies (Zhang et al., 2017; Cheng et al., 2020). The bacterial diversity exhibited a consistent association with the pH gradient, which can be attributed to the narrower pH optima of specific bacteria (Lagos et al., 2016; Rousk et al., 2010). Moreover, the increased availability of nutrients resulting from fertilizer stimulated bacterial growth and contributed to increased bacterial diversity (Cheng et al., 2020). However, the low SOC content resulting from high-level fertilizer constrains bacterial growth (Demoling et al., 2007).

**Figure 8:**
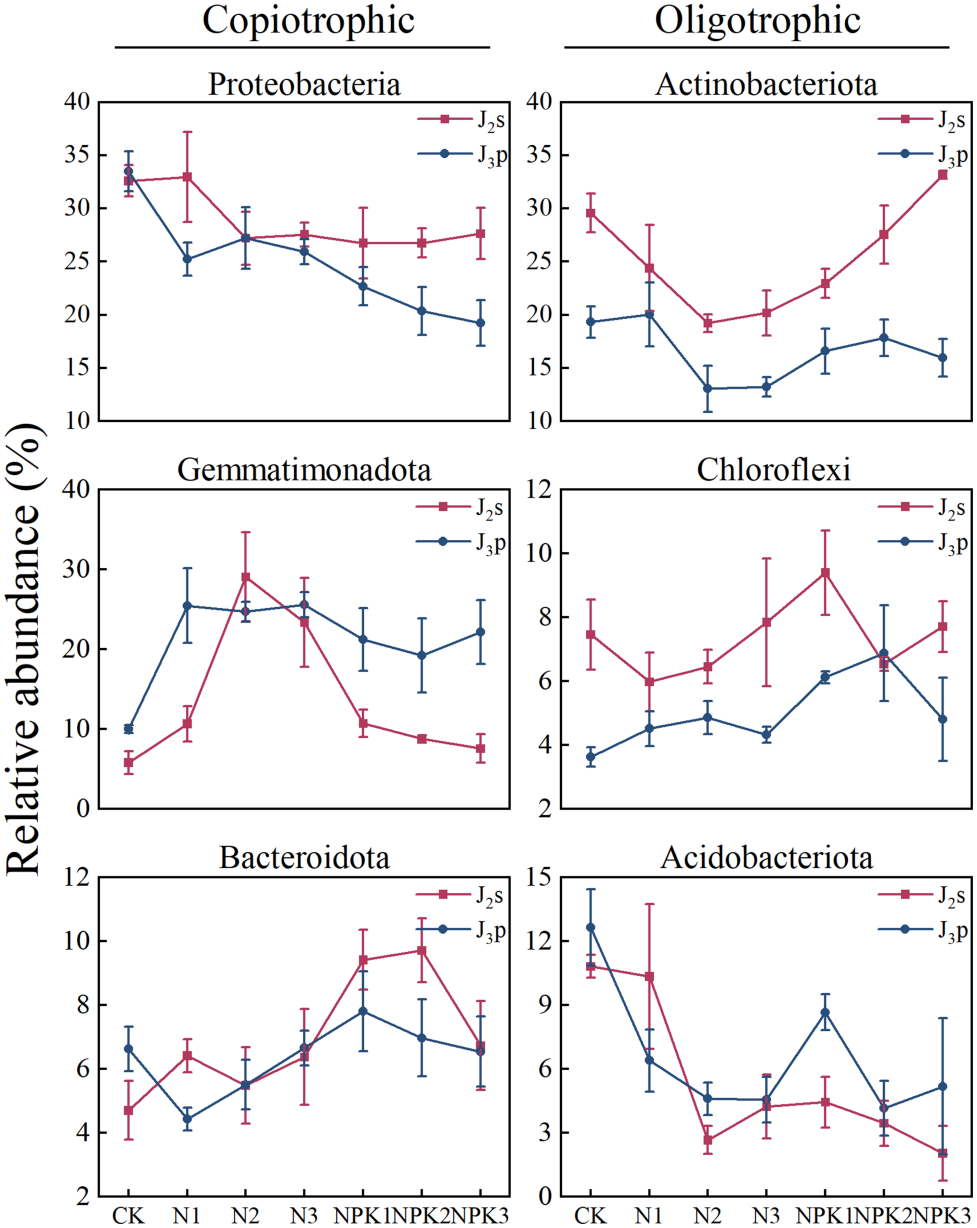
The relative abundance of bacteria classified into copiotrophoc and oligotrophic groups at the phylum level under different treatments.

**Figure 9:**
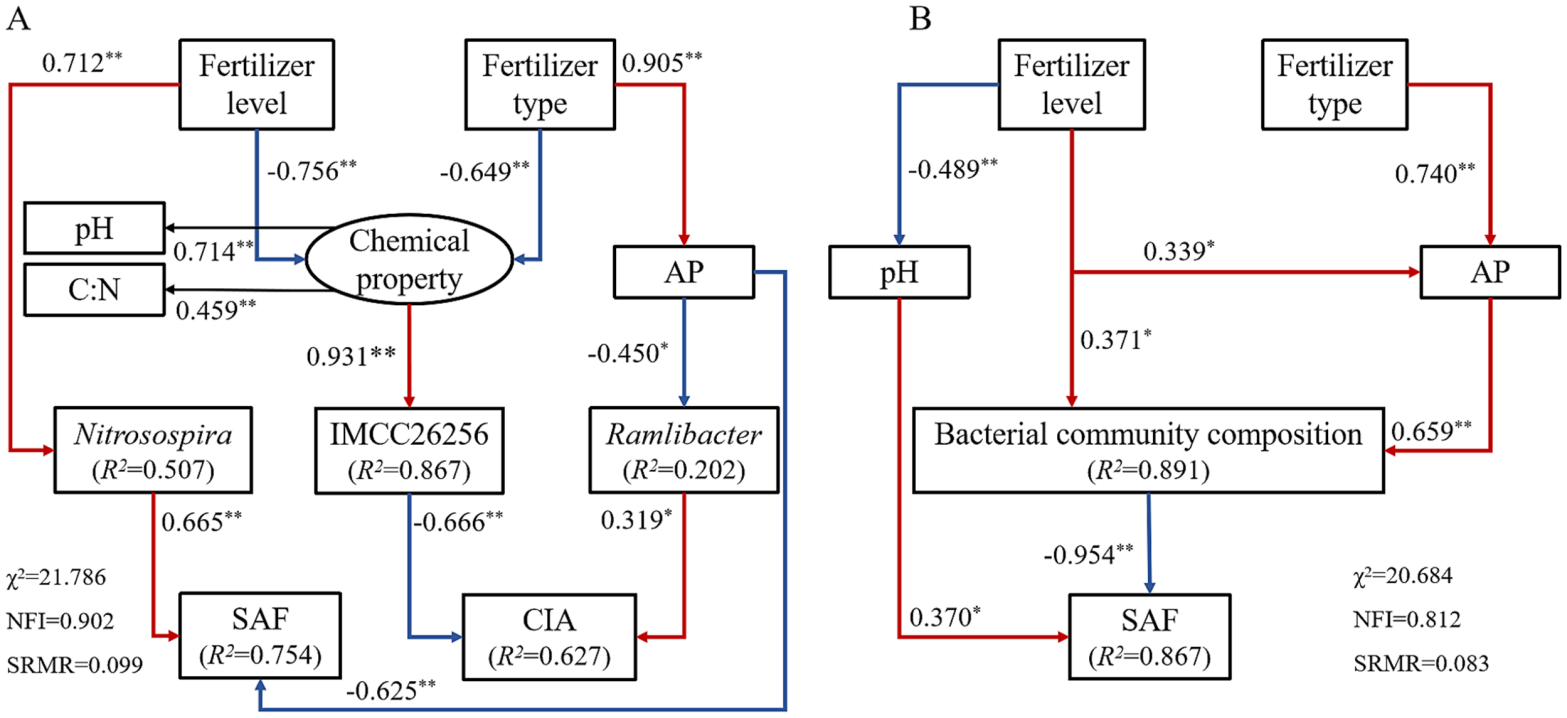
Structure equation models (SEMs) of the relationships between the chemical properties, relative abundance of dominant genus, bacterial abundance, diversity and community composition. **(A)** J_2_s purple parent rock. **(B)** J_3_p purple parent rock. Lines in red and blue color with arrows indicate significant positive and negative paths, respectively. Numbers on the lines indicate the standard path coefficients, * and ** indicate significances at *p <* 0.05 and *p <* 0.01. *R^2^* values represent the proportions of variance explained by relationships with other variables.

According to NMDS (Figure 3), differences in the composition of bacterial communities have also been observed under different fertilizer treatments, which has been confirmed in previous studies (Yuan et al., 2013; Leff et al., 2015; Cheng et al., 2020). The dominant phyla with relative abundances greater than 1% for all samples were Proteobacteria, Actinobacteriota, Gemmatimonadota, Bacteroidota, Chloroflexi, and Acidobacteriota. The relative abundance of these dominant phyla varied under the different fertilizer treatments (Figure 1). Fertilization-induced changes in bacterial community composition can be explained by the co-nutrient hypothesis: nutrient enrichment favors copiotrophic bacteria colonization, while oligotrophic ones may be unaffected or negatively affected (Dai et al., 2017; Fierer et al., 2007). An examination of the relative abundances of the dominant phyla (Figure 8) revealed that Gemmatimonadota and Bacteroidota (copiotrophic groups) generally tended to increase under fertilizer application. Conversely, Actinobacteriota and Acidobacteriota (oligotrophic groups) displayed an overall decreasing trend under fertilizer application, whereas the relative abundance of Chloroflexi (oligotrophic group) did not significantly differ among the treatments. Surprisingly, the relative abundance of Proteobacteria (copiotrophic group) decreased under fertilizer application, particularly in response to NPK fertilizer treatments. Proteobacteria are known to thrive in nutrient-rich environments, primarily consuming labile organic matter (Demoling et al., 2007). Therefore, despite the significant increase in nutrient availability resulting from fertilizer application, the concurrent decrease in the SOC content renders the environmental conditions unfavorable for the growth and reproduction of Proteobacteria. This finding suggested that, in addition to nutrient preferences, different bacteria have distinct limiting conditions for each nutrient. Redundancy analysis (RDA) of the dominant genera (Supplementary Table S4) revealed that the contents of AP and AN and the pH of the weathered products were critical factors influencing bacterial growth under fertilization. Notably, the AP content had a stronger effect on the bacterial community composition than did the other chemical factors. These findings suggest that fertilizer application mitigates bacterial phosphorus limitation and influences bacterial community composition by increasing phosphorus effectiveness (Liu et al., 2020). This finding was further validated via SEM (Figure 9).

These alterations in biochemical properties that are consequent to fertilizer application affect the weathering processes and velocity of purple parent rocks through multiple pathways. Compared with the CK treatment, fertilizer application stimulated chemical weathering in J_2_s (Figure 5). The *CIA* of J_2_s was correlated with pH, SOC content, C:N, bacterial community composition, and specific bacteria (Figure 7). SEM revealed that the pH, C:N, AP content, relative abundance of IMCC26256, and relative abundance of *Ramlibacter* were key factors influencing the chemical weathering of J_2_s (Figure 9). Specifically, the pH and C:N of weathered products under different fertilizer conditions were the most important drivers that interactively controlled chemical weathering through the influence of the relative abundance of IMCC26256. IMCC26256 is significantly and positively correlated with the N_2_O index (Sui et al., 2020), indicating the involvement of IMCC26256 in the nitrogen cycling process of weathered products, which is proven to be correlated with weathering processes. Additionally, AP regulated J_2_s chemical weathering through *Ramlibacter*. *Ramlibacter* possesses genes associated with the phosphorus scavenging pathway, which are involved in high-affinity phosphate acquisition and storage of polyphosphate particles and play crucial roles in residual phosphorus mineralization (Props et al., 2019; Zhang et al., 2021). The phosphorus solubilization process can facilitate the release of Ca and Mg, thereby impacting mineral weathering (Li et al., 2023). The input of AP resulting from fertilizer application disrupts the P balance regulated by *Ramlibacter* and inhibits the growth and reproduction of *Ramlibacter*, consequently affecting the weathering process.

Fertilizer application enhanced the physical weathering of J_2_s, except for the NPK fertilizer treatments at the 100 and 200% levels (Figure 6). Correlation analysis revealed that the contents of TN and AP, the bacterial community composition, and specific bacteria were correlated with the *SFR* (Figure 7). SEM revealed that the level of fertilizer had a positive effect on *Nitrosospira*, which plays a role in physical weathering processes (Figure 9). *Nitrosospira* belongs to the group of ammonia-oxidizing bacteria (AOB), and its ammonia oxidation activity represents the rate-limiting step in nitrification (Huang et al., 2023). Previous studies have demonstrated that the H^+^ produced during nitrification under fertilization undergoes ion exchange reactions with minerals within rock crystals, leading to cavity enlargement, pore unclogging, and enhanced rock disintegration and destruction (Li et al., 2021; Song et al., 2017a). Furthermore, fertilizer type impacts the AP content, significantly influencing the physical weathering of J_2_s. Similar to the mediation mechanism by which *Ramlibacter* mediates *CIA* through AP, the process of P dissolution promotes mineral weathering. However, the introduction of excessive amounts of AP can inhibit this process. This observation helps explain the disparity in the degree of physical weathering between the two different types of fertilization.

Compared with that of J_2_s, the chemical weathering of J_3_p was not sensitive to fertilizer application. Under the same fertilizer type, chemical weathering degree of J_3_p tended to decrease with increasing fertilizer level. This finding aligns precisely with the results derived from the incubation experiments focused on the influence of nitrogen fertilizer on weathering (Li et al., 2023). The *CIA* was not correlated with any of the factors except the relative abundance of Subgroup_7. According to the SEM (Figure 9), an effective pathway to influence chemical weathering was not formed by the given factors, which may have occurred because the influence of fertilization on chemical weathering factors was much lower than the influences of external environmental factors, such as rainfall and temperature. These changes did not reach the threshold for causing changes in chemical weathering.

In contrast, the NPK fertilization treatments significantly enhanced the physical weathering of J_3_p (Figure 6). Correlation analysis revealed that the *SFR* of J_3_p was associated with the CEC, the contents of TP and available nutrients, the bacterial community composition, and the abundances of specific bacteria (Figure 7). SEM revealed that the impact of fertilizer application on the physical weathering of J_3_p can be divided into two pathways (Figure 9). First, the level of fertilizer had a negative effect on the pH, directly influencing physical weathering. Previous studies have demonstrated that H^+^ input accelerates weathering. However, J_3_p exhibited the opposite trend due to its high pH value and the neutralizing effect of its large amount of CaCO_3_, which resulted in minimal influence of fertilizer application on the pH. Consequently, although the SEM indicated a positive feedback effect on physical weathering, the actual path coefficient was small. Second, fertilizer application indirectly affected the bacterial community composition through the AP and directly affected the bacterial community composition through the fertilization level, thereby influencing physical weathering in J_3_p. RDA revealed that AP was the primary driver of bacterial community composition Multiple studies have shown that nutrient inputs and associated environmental changes can alter bacterial community composition (Leff et al., 2015; Cheng et al., 2020; Zi et al., 2023). Recent findings from functional gene screening studies further support these results, indicating that the distribution and function of weathering-associated bacteria are strongly influenced by external factors such as pH, nutrient effectiveness, and carbon and nitrogen sources (Chen et al., 2022; Epihov et al., 2021). These findings align with the results obtained in this study.

In addition, the intrinsic properties of the parent rock (mineral composition and resistance to weathering) are major factors in the selection bias of weathering bacteria. For example, *Burkholderia*, which has an excess of negatively charged surface groups and phosphate groups, is more inclined to adsorb to orthoclase, which has a less negatively charged surface (Uroz et al., 2015). This explains the difference in bacterial weathering between the two parent rock types under fertilizer application.

## 5 Conclusion

Fertilizer application altered the bacterial community, with increasing fertilizer levels leading to decreased bacterial diversity and abundance and changes in community composition of weathered products. The two types of parent rock have different weathering characteristics and sensitivities to fertilizer types. J_2_s manifested more potent chemical weathering, whereas J_3_p exhibited relatively preponderant physical weathering. Overall, fertilizer application enhanced weathering of the purple parent rock.

Through correlation analysis and structural equation models, the mechanisms of fertilization impacting the weathering of purple parent rocks were revealed: (1) Fertilizer application exerts indirect effects on physicochemical weathering through the modulation of chemical properties (pH, C:N and AP content), which are mediated by specific bacteria (IMCC26256 and *Ramlibacter*), and the overall bacterial community composition of weathered products. (2) Fertilizer application directly influences physical weathering by directly modifying chemical properties (pH and AP content). (3) Fertilizer application directly impacts physical weathering by altering the relative abundance of specific bacteria (*Nitrosospira*).

This study explored the effects of fertilizer application on bacteria and weathering. However, in reality, the influence of plants on rock weathering under fertilization cannot be ignored. Moreover, plants and microorganisms’ metabolic activities interact. Future studies should comprehensively consider their combined effect on rock weathering to dissect fertilization’s impact. Consequently, it can provide a novel approach for the research on the sustainable development, carbon cycling, and climate regulation of agricultural areas.

## Data Availability

The datasets presented in this study can be found in online repositories. The names of the repository/repositories and accession number(s) can be found in the article/Supplementary material.
