# Supplementary material for: Effects of fertilizer application on the bacterial community and weathering characteristics of typical purple parent rocks
Source: Front Microbiol. 2025 Jan 7;15:1514646. doi: 10.3389/fmicb.2024.1514646 (PMC11752876; doi:10.3389/fmicb.2024.1514646)
Supplement: Supplementary file 1 [file Data_Sheet_1.docx]

Supplementary Material

# Supplementary Figures and Tables

## Supplementary Tables

**Table S1.** The mineral composition of purple parent rock before leaching experiment.

| Parent rock | Mineral content（%） | | | | | | | | | Relative content of clay minerals（%） | | | |
| --- | --- | --- | --- | --- | --- | --- | --- | --- | --- | --- | --- | --- | --- |
|  | Qtz | Kfs | Ab | Lau | Ank | Dol | Cal | Hem | Clays | I/S | It | Kao | Chl |
| J2s | 32.25 | 2.75 | 18.19 | 0 | 0 | 0 | 1.4 | 2.5 | 42.91 | 58.39 | 16.89 | 10.21 | 14.51 |
| J3p | 32.73 | 1.83 | 11.48 | 0 | 0 | 5.21 | 15.29 | 0.65 | 32.81 | 69.5 | 21.34 | 5.17 | 3.99 |

**Table S2.** The main oxide changes of purple parent rocks before and after the leaching experiment (%).

| Parent rock | Oxide | CK | N1 | N2 | N3 | NPK1 | NPK2 | NPK3 | Original rock |
| --- | --- | --- | --- | --- | --- | --- | --- | --- | --- |
| J2s | SiO2 | 60.00±0.16 | 60.13±0.12 | 60.17±0.05 | 60.00±0.08 | 60.07±0.25 | 59.57±0.25 | 59.57±0.19 | 60.81±1.34 |
|  | Fe2O3 | 8.52±0.02 | 8.58±0.12 | 8.41±0.06 | 8.48±0.06 | 8.40±0.13 | 8.56±0.23 | 8.55±0.04 | 7.59±0.11 |
|  | Al2O3 | 19.97±0.09 | 20.03±0.17 | 20.10±0.08 | 20.27±0.09 | 19.87±0.05 | 19.93±0.12 | 20.10±0.08 | 20.01±1.41 |
|  | K_2_O | 3.85±0.01 | 3.87±0.02 | 3.85±0.03 | 3.92±0.04 | 3.82±0.03 | 3.90±0.06 | 3.91±0.02 | 3.53±0.03 |
|  | CaO | 1.94±0.09 | 1.75±0.09 | 1.74±0.02 | 1.60±0.01 | 1.88±0.02 | 1.93±0.05 | 1.96±0.13 | 1.91±0.22 |
|  | Na2O | 1.22±0.03 | 1.17±0.01 | 1.17±0.03 | 1.18±0.02 | 1.14±0.04 | 1.15±0.05 | 1.15±0.03 | 1.28±0.01 |
|  | MgO | 3.04±0.02 | 3.06±0.04 | 3.08±0.02 | 3.14±0.04 | 3.10±0.01 | 3.05±0.06 | 3.00±0.03 | 3.43±0.09 |
|  | TiO2 | 0.81±0.01 | 0.84±0.04 | 0.87±0.04 | 0.85±0.01 | 0.85±0.02 | 0.85±0.02 | 0.82±0.01 | 0.88±0.02 |
|  | MnO | 0.11±0.00 | 0.10±0.00 | 0.10±0.00 | 0.09±0.01 | 0.10±0.01 | 0.10±0.01 | 0.11±0.01 | 0.09±0.00 |
| J3p | SiO2 | 57.65±0.45 | 57.05±0.95 | 57.30±0.10 | 57.95±0.15 | 57.93±0.40 | 57.53±0.58 | 56.97±0.39 | 57.00±0.24 |
|  | Fe2O3 | 6.30±0.22 | 6.40±0.36 | 6.30±0.14 | 6.05±0.16 | 6.04±0.14 | 6.08±0.34 | 6.44±0.12 | 5.81±0.09 |
|  | Al2O3 | 16.40±0.10 | 16.40±0.40 | 16.45±0.15 | 16.10±0.20 | 15.65±0.55 | 15.93±0.40 | 16.03±0.21 | 15.10±0.19 |
|  | K_2_O | 3.48±0.04 | 3.55±0.10 | 3.51±0.09 | 3.41±0.05 | 3.44±0.07 | 3.45±0.11 | 3.60±0.05 | 3.16±0.05 |
|  | CaO | 10.65±0.05 | 11.10±0.10 | 11.05±0.35 | 11.00±0.60 | 11.18±0.56 | 10.43±1.14 | 10.83±0.33 | 13.2±0.29 |
|  | Na2O | 0.92±0.02 | 0.88±0.07 | 0.91±0.04 | 0.94±0.01 | 0.96±0.02 | 0.95±0.05 | 0.89±0.02 | 1.01±0.03 |
|  | MgO | 3.17±0.04 | 3.21±0.04 | 3.18±0.01 | 3.15±0.00 | 3.06±0.03 | 3.13±0.03 | 3.09±0.06 | 3.22±0.03 |
|  | TiO2 | 0.81±0.04 | 0.84±0.04 | 0.83±0.03 | 0.84±0.01 | 0.79±0.02 | 0.85±0.02 | 0.85±0.01 | 0.87±0.02 |
|  | MnO | 0.11±0.00 | 0.12±0.01 | 0.12±0.01 | 0.11±0.01 | 0.11±0.01 | 0.12±0.01 | 0.10±0.00 | 0.15±0.01 |

* CK=No fertilizer; N1=280 kg·ha^-1^·a^-1^N; N2=560 kg·ha^-1^·a^-1^N; N3=840 kg·ha^-1^·a^-1^N; NPK1=N-P_2_O_5_-K_2_O: 280-180-72 kg·ha^-1^·a^-1^; NPK2=N-P_2_O_5_-K_2_O: 560-360-144 kg·ha^-1^·a^-1^; NPK3=N-P_2_O_5_-K_2_O:kg·ha^-1^·a^-1^.

**Table S3.** The chemical properties of purple parent rocks before leaching experiment.

| Parent rock | pH | CEC | SOC | TN | TP | TK | AN | AP | AK |
| --- | --- | --- | --- | --- | --- | --- | --- | --- | --- |
| J_2_s | 10.18 | 16.66 | 0.27 | 0.02 | 0.08 | 2.93 | 10.68 | 3.86 | 121.2 |
| J_3_p | 9.14 | 15.42 | 0.14 | 0.02 | 0.07 | 2.62 | 10.64 | 7.50 | 66.23 |

*CEC(cmol/kg)—Cation exchange capacity；TN(\%)—Total nitrogen; SOC(\%)—Soil organic carbon; TP(\%)—Total phosphorus; TK(\%)—Total potassium; AN(\%)—Available nitrogen; AP(\%)—Available phosphorus; AK(\%)—Available potassium.

**Table S4.** Redundancy analysis (RDA) of the effect of different chemical properties on the bacterial community composition under different treatments.

| Parent rock | Factors | Explains(%) | Contribution(%) | pseudo-F | p_value |
| --- | --- | --- | --- | --- | --- |
| J_2_s | AP | 23.6 | 40.3 | 5.9 | 0.014 |
|  | AN | 13.8 | 23.6 | 4.0 | 0.030 |
|  | TN | 4.4 | 7.4 | 1.3 | 0.306 |
|  | TP | 3.3 | 5.6 | 1.0 | 0.298 |
|  | pH | 5.0 | 8.4 | 1.5 | 0.214 |
|  | TK | 3.6 | 6.1 | 1.1 | 0.300 |
|  | AK | 2.0 | 3.4 | 0.6 | 0.576 |
|  | CN | 1.3 | 2.3 | 0.4 | 0.716 |
|  | CEC | 0.6 | 1.0 | 0.1 | 0.940 |
| J_3_p | AP | 32.6 | 45.2 | 9.2 | 0.002 |
|  | pH | 9.8 | 13.6 | 3.1 | 0.038 |
|  | TN | 6.7 | 9.3 | 2.2 | 0.090 |
|  | TK | 2.9 | 4.1 | 1.0 | 0.374 |
|  | CEC | 7.2 | 10.0 | 2.7 | 0.066 |
|  | TP | 3.5 | 4.8 | 1.3 | 0.302 |
|  | AN | 4.6 | 6.4 | 1.8 | 0.146 |
|  | AK | 0.9 | 1.2 | 0.3 | 0.790 |

*CEC(cmol/kg)—Cation exchange capacity；TN(\%)—Total nitrogen; SOC(\%)—Soil organic carbon; TP(\%)—Total phosphorus; TK(\%)—Total potassium; AN(\%)—Available nitrogen; AP(\%)—Available phosphorus; AK(\%)—Available potassium.

## Supplementary Figures


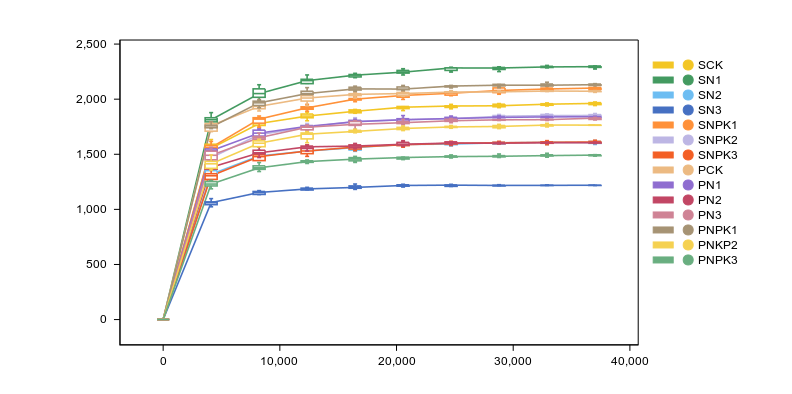


**Figure S1.** Rarefaction Curve of bacteria under different treatments. S— J_2_s purple parent rock, and P—J_3_p purple parent rock.
